# Histological Subtypes of Bladder Cancer: Epidemiology, Molecular Biology, and Clinical Implications

**DOI:** 10.3390/cancers18132174

**Published:** 2026-07-07

**Authors:** Belén Mora-Garijo, Syed Rahman, Hongzhi Xu, Jon Chatzkel, G. Daniel Grass, Philippe E. Spiess, Roger Li

**Affiliations:** 1 Department of Urology, Medstar Georgetown University Hospital, Washington, DC 20007, USA; 2 Department of Urology, Yale School of Medicine, New Haven, CT 06510, USA; syed.rahman@yale.edu; 3 Department of Genitourinary Oncology, Moffitt Cancer Center, Tampa, FL 33612, USA; hongzhi.xu@moffitt.org (H.X.); jon.chatzkel@moffitt.org (J.C.); daniel.grass@moffitt.org (G.D.G.); philippe.spiess@moffitt.org (P.E.S.)

**Keywords:** bladder cancer, urothelial carcinoma, histologic subtypes of bladder cancer, variant histology

## Abstract

Histological subtypes of bladder cancer are generally associated with more aggressive disease, higher stage at presentation, and worse prognosis compared to conventional urothelial carcinoma. Recent updates in classification systems have further refined the distinction between true histologic subtypes and divergent differentiation, underscoring the complexity of these tumors. Emerging molecular profiling studies have identified subtype-specific genomic alterations, including ERBB2 amplification in micropapillary subtype carcinoma, CDH1 loss in plasmacytoid subtype carcinoma, and TP53 and RB1 co-alterations in neuroendocrine bladder cancer, which may contribute to differences in tumor biology and therapeutic response. Despite these advances, the histological subtypes of bladder cancer remain underrepresented in prospective clinical trials, leading to significant gaps in evidence-based management. Treatment responses vary widely across subtypes, with some demonstrating sensitivity to platinum-based chemotherapy or immunotherapy, while others appear less responsive to conventional approaches. This review summarizes the current understanding of the epidemiology, molecular landscape, and clinical behavior of major bladder cancer subtypes and highlights emerging opportunities for personalized treatment strategies, biomarker-driven therapies, and more inclusive clinical trial design to improve outcomes in this high-risk population.

## Simple Summary

Bladder cancer includes several less common subtypes that differ from the typical form of the disease in how they behave and respond to treatment. These histological subtypes of bladder cancer are often more aggressive, are diagnosed at a later stage, and may not respond as well to standard therapies. Despite this, they have historically been underrepresented in clinical studies, making it difficult for clinicians to determine the most effective treatment strategies. In this review, we summarize the current understanding of these subtypes, including their biological features and how they respond to different treatments. We also highlight recent advances in molecular research that may help explain why these cancers behave differently. A better understanding of these subtypes may support the development of more personalized treatment approaches and improve outcomes for patients with these high-risk forms of bladder cancer.

## 1. Introduction

The subtypes of bladder cancer (SBC) are a heterogenous group of bladder cancers that exhibit distinct histological features [1]. SBC is generally considered more aggressive than standard urothelial bladder cancer with clinical evidence suggesting specific underlying molecular differences correlating to the individual subtypes [2]. Historically, most clinical trials evaluating the treatment of high-risk non-muscle invasive bladder cancer (NMIBC) and muscle invasive bladder cancer (MIBC) have incorporated few patients with SBC, leading to uncertainty regarding their optimal treatment strategies. Of note, a recent phase II clinical trial investigating the treatment of non-urothelial and metastatic carcinoma patients treated with durvalumab and tremelimumab stratified by individual subtype demonstrated no observed response in 13 patients with neuroendocrine, adenocarcinoma, and squamous differentiation [3]. Studies such as these suggest the growing need to further elucidate molecular drivers and targets for these SBCs.

Recently, the nomenclature for SBC has changed, with the World Health Organization (WHO) recognizing a distinction between divergent differentiation, such as urothelial carcinoma with squamous or glandular differentiation reflecting non-urothelial lineages, and histologic subtypes, such as micropapillary, plasmacytoid, sarcomatoid, and neuroendocrine carcinoma, which retain urothelial lineage with distinct architectural features (Table 1) [4], all of which are considered high-grade tumors.

Ultimately, these histologic subtypes and divergent differentiations (for simplicity, they will jointly be referred to as SBC) present at higher clinical stages and have a greater propensity for progression [5]. More importantly, their underlying genomic differences may correlate to their varying clinical phenotypes and may offer distinct therapeutic targets for future therapeutic development [6,7,8]. In this review, we examine the molecular underpinnings, histopathologic features, and clinical behavior of these subtypes. Our review is novel in its emphasis on the molecular characteristics of these subtypes while also synthesizing literature-based insights into treatment sequencing in the setting of poorly defined management algorithms. Specifically, we will focus on micropapillary, plasmacytoid, sarcomatoid, glandular differentiation and neuroendocrine, as well as squamous differentiation and squamous cell carcinoma.

## 2. Materials and Methods

This narrative review summarizes the epidemiology, molecular biology, and clinical implications of several histologic subtypes of bladder cancer. The 2022 World Health Organization (WHO) classification of urinary tract tumors served as the framework for defining and categorizing the subtypes discussed. The review focuses on the most prevalent SBC, particularly those associated with the poorest clinical outcomes. Additionally, we acknowledge that other clinically relevant bladder cancer variants such as nested, lymphoepithelioma-like, clear-cell, giant-cell, lipid-rich, and trophoblastic differentiation are not included for the purposes of this manuscript.

A literature search was performed primarily using PubMed/MEDLINE, employing the search terms “bladder cancer,” “subtypes,” and “variant histology,” combined with the name of each individual histological subtype (e.g., “micropapillary,” “plasmacytoid,” “sarcomatoid,” “neuroendocrine,” “small cell carcinoma,” “adenocarcinoma,” “glandular/squamous/neuroendocrine differentiation,” and “squamous cell carcinoma”). The search concluded in May 2026, including studies ranging in publication date from 2009 to 2025. Peer-reviewed, English-language original studies, with a focus on large retrospective cohorts, clinical trials, genomic analyses, and current clinical practice guidelines were included. Non-English publications and studies not pertaining to histologic subtypes of bladder cancer were excluded. The search was conducted by two authors (B.M.-G. and S.R.), with additional relevant publications identified and contributed by the senior author (R.L.).

## 3. Micropapillary Subtype

Micropapillary subtype carcinoma (MPC) is characterized by small nests of tumor cells without fibrovascular cores surrounded by lacunae empty spaces. However, similar findings of papillary extensions can be identified in conventional urothelial carcinoma with retraction artifacts. Therefore, additional studies have demonstrated moderate interobserver variability in the diagnosis of MPC, posing a challenge for pathologic diagnosis [9]. In a retrospective analysis of all cases of MPC in the National Cancer Database (NCDB), it was identified that MPC is an aggressive disease presenting often at advanced stages and associated with a high propensity for disease progression. Less than 41% of patients were cT1 and the majority were cT2+ with MPC being more likely to present with clinically node positive disease (9% compared to 2% in pure conventional urothelial carcinoma). Ultimately those that were treated with radical cystectomy (RC) had a median overall survival (OS) of 40 months from the time of surgery [10]. Moreover, the aggressive nature of MPC has impacted urologists’ perception of the disease and influenced their treatment algorithm. In a 2015 survey sent to members of SUO regarding the management of MPC, 73% of urologic oncologists thought that MPC did not respond to cisplatin-based chemotherapy with 50% also stating that the subtype does not respond to BCG. Ultimately, 47% believed that cT2 MPC should be managed with upfront cystectomy rather than undergoing neoadjuvant chemotherapy, thus highlighting both the understanding of the disease’s aggressive nature as well as its non-response to standard therapeutic measures [11].

From the molecular perspective, a recent study utilized next-generation sequencing to analyze 99 MPC specimens (74 primary and 25 metastatic) [12]. The most common gene mutations identified were *TP53*, *TERT*, *ARID1A*, *ERBB2*, and *RB1*. Additionally, *ERBB2* amplification and *HER2* overexpression correlated with worse OS. Differences in the genomic landscape associated with *ERBB2*-mutated compared to non-mutated MPCs were also described, with *ERBB2*-mutated tumors exhibiting more prevalent alterations in *KMT2D*, *RB1*, and *MTAP* genes, several of which have previously been linked to worse survival [12]. Additionally, in the NMIBC space, Bellmunt et al. performed whole transcriptome sequencing of 23 high-grade T1 MPCs and pure urothelial carcinoma (UC) patients, identifying differential expression in over 3000 genes. Three genes: *CD36*, *FABP3*, *RAETE1* were associated with shortened time to progression, suggesting elevated biological aggressiveness associated with the expression of these three genes [13].

Clinically, the treatment strategies used for MPC have been variable. For NMI MPC, a retrospective analysis of 72 cT1N0M0 tumors demonstrated a 5-year cancer-specific survival (CSS) of 100% in those undergoing RC compared to 60% in those initially undergoing BCG treatment, suggesting a benefit to early surgical extirpation [14]. In regard to MIBC, there has been varying data regarding chemosensitivity. Amin et al. investigated 103 patients with resectable disease, showing complete pathologic response (pT0N0) in 52% who received neoadjuvant cisplatin-based chemotherapy (NAC) vs. 19% in those who did not [15]. Similarly, Meeks et al. demonstrated in an analysis of 42 patients with cT2 disease that pathologic complete response was achieved in 13 patients who received NAC (45%) compared to 2 (13%) undergoing upfront RC [16]. Ultimately, a retrospective analysis of the NCDB by Rahman et al. demonstrated that NAC use was associated with greater odds of pT0 (9.64 [7.62–12.82], *p* < 0.001), lower odds of pN+, and pathologic downstaging in all stages cT2+. However, no difference in OS was identified [6]. *ERBB2* amplification and constitutive *HER2* receptor activation preferentially seen in MPC drive downstream cell signaling (PI3L/AKT/MAPK) which promotes tumor cell proliferation and survival while conferring resistance to apoptotic stimuli. This may partially explain the variable chemosensitivity observed in MPC and supports investigations of HER2-directed agents. Ultimately, this demonstrates that the data regarding chemosensitivity in muscle invasive MPC is largely variable due to likely retrospective analysis and requiring additional high quality analysis and studies for further definitive conclusions to be drawn from.

## 4. Plasmacytoid Subtype

Plasmacytoid subtype (PCUC) is characterized by single infiltrating cells with or without cytoplasmic vacuoles characterized with known discohesive growth that can easily spread across tissue planes. It is characterized by an OS of 30% and represents 1–2% of all MIBC diagnoses with most data coming from small retrospective single center analyses [17]. From a retrospective analysis of 53 MD Anderson patients with localized PCUC, only 28% were alive following a median of 36-month follow-up. Staging was as follows: cT2N0 in 22 (39.3%), cT3N0 in 15 (26.8%), cT4N0 in 13 (23.2%), and cN1 in six patients (10.7%). In the group that underwent upfront RC, four patients (7.2%) had pT0N0 while 22 (52.4%) had pN+ disease at the time of surgery. NAC was not associated with improved CSS or metastasis-free survival (MFS) (*p* = 0.61, *p* = 0.54) for patients with cT2+ disease. Overall, 42 patients developed disease recurrence or metastasis, of whom 16 did not receive subsequent therapy, seven received immunotherapy (IO) and chemotherapy, and 19 received salvage chemotherapy alone. Salvage immunotherapy in these patients significantly reduced the risk of cancer-associated death (HR 0.11, *p* < 0.001) [8]. Additionally, in a retrospective analysis of NCDB and patients treated at Yale, Cox proportional hazards model incorporating clinical stage and age revealed no correlation between NAC and survival [7].

PCUC is characterized by loss of E cadherin protein expression (a cell adhesion protein whose loss allows increased cellular motility and metastases) [18]. A small study investigating whole exome analysis of six tumors, PCUC was characterized by *CDH1* hypermethylation, leading to loss of E cadherin expression. Other mutations commonly identified include *TP53*, *FGFR3*, *RB1*, *PIK3CA*, and *TERT* promoter [18]. Proteins in the mTOR pathway also have been suggested to be of clinical relevance in PCUC with 19 histopathologic cases demonstrating 100% of cases positive for some degree of cytoplasmic *PTEN* expression and phospho-AKT [19]. Additional proteins that have been identified to have increased expression in PCUC include HER2 (with retrospective analyses suggesting expression in 25–83% of PCUC cases) and Nectin-4 (positive immunohistologic staining in 63% of cases). Viable targets such as Nectin-4 (enfortumab) and HER2 (trastuzumab) should be further investigated [2]. Ultimately, the majority of mutations characteristic to PCUC, particularly E-cadherin loss, drive an epithelial-to-mesenchymal transition (EMT) dysregulation, conferring the discohesive, infiltrative growth pattern characteristic of PCUC and its propensity for peritoneal spread. Mechanistically, this EMT state is associated with reduced chemosensitivity, consistent with the lack of NAC benefit observed clinically [8].

## 5. Carcinoma with Glandular Differentiation and Pure Adenocarcinoma

Carcinoma with glandular differentiation is characterized as a spectrum ranging from urothelial carcinoma with glandular differentiation to pure adenocarcinoma of the urachus or the bladder. A retrospective study of 37 patients with bladder adenocarcinoma (AC), 46 with urachal AC, 84 with UC with glandular differentiation, and 1049 with conventional UC treated at Memorial Sloan Kettering Cancer Center (MSKCC) demonstrated that patients with primary bladder AC and urachal AC were more likely to have locally advanced disease (pT3-4; 57% and 63%, respectively) [20]. Ultimately, when adjusted for age, no significant differences in recurrence or CSS was observed comparing glandular differentiation to UC overall. Among 29 patients with UC with glandular differentiation who received NAC, five (17%) demonstrated pT0N0 and eight (28%) had pathologic downstaging (≤pT1N0), compared with 48 of 189 patients (25%) with pT0N0 and 114 patients (60%) with pathologic downstaging (≤pT1N0) in the UC group. Moreover, this suggests that along the glandular spectrum there exists chemosensitivity to NAC, but perhaps to a lesser degree compared to conventional UC [20].

Moreover, primary AC of the bladder demonstrated high rates of oncogenic alterations in *TP53*, *KRAS*, and *PIK3CA* (similar to rates observed in colorectal adenocarcinoma). Additionally, the urachal AC demonstrated high rates of *SMAD4* alterations (24%), with rates higher than those observed in colorectal adenocarcinoma. Deleterious DNA damage response (DDR) gene alterations had been previously associated with improved outcomes in the patients with MIBC and highlighted responsiveness to platinum-based chemotherapy administration [21]. In concordance with the glandular differentiation and adenocarcinoma spectrum, primary bladder or urachal AC rarely demonstrated DDR mutations, whereas UC with glandular differentiation had similar rates of DDR mutations compared to pure UC, suggesting a similar genomic profiles between pure UC and UC with glandular differentiation, but distinct from pure adenocarcinoma [20].

Ultimately, similar strategies are used to treat patients with UC with glandular differentiation and AC as those with conventional UC. In a retrospective analysis of the NCDB investigating the response of histologic subtypes to NAC, Vetterlein et al. demonstrated NAC was able to decrease the frequency of non-organ-confined disease without prolonging OS in a subpopulation of 357 adenocarcinomas. Taken together with the MSKCC retrospective series, this study suggests a role for NAC in the treatment of glandular differentiation, even though there is less chemosensitivity in the pure AC cohort compared to conventional UC [22].

## 6. Neuroendocrine Carcinoma

Neuroendocrine bladder cancer (NEBC) represents ~1% of urinary bladder cancer, including primarily small cell neuroendocrine carcinoma and large cell neuroendocrine carcinoma. This section will focus on small cell neuroendocrine bladder cancer (SCNC) given that it represents the most common form of NEBC [23]. SCNC is typically found in male patients over the age of 60. SCNC is associated with a high metastatic potential and a 5-year survival rate of less than 10% [24].

Histologically, SCNC is defined by features such as sheets and nests of tumor cells with scant cytoplasm, speckled nuclei, and indistinct nucleoli, nuclear moldings, numerous mitoses and coagulative necrosis. Immunohistochemical staining with classic neuroendocrine biomarkers, including chromogranin A, synaptophysin, INSM1, and CD56, assist in the diagnosis of NEBC [25]. Approximately 40% of SCNC cases exhibit mixed histological components of small cell and conventional UC, suggesting a molecular continuum and shared common clonal origin between UC and SCNC. This theory is further substantiated by research demonstrating shared mutations between SCNC and UC. Chang et al. compared 87 SCNC, 303 high-grade UC, and 149 small cell lung cancer (SCLC) cases and found a similar tissue-of-origin specific mutational pattern of somatic *RB1* and *TP53* driver mutations in SCNC and UBC, which were absent in SCLC [23,24].

The inactivation of *TP53* and *RB1*, as well as high prevalence of APOBEC-driven mutations serve as hallmarks for NEBC [23]. For instance, a study of 61 SCNC patients from MSKCC found 90% of tumors harbored alterations of *TP53* and *RB1*, with co-alterations occurring in 80% [26]. This dual inactivation not only enables unconstrained tumor cell proliferation but has been shown to facilitate neuroendocrine transdifferentiation through activation of lineage-specific transcription factors such as ASCL1 and NEUROD1. In addition, *TERT* promoter mutations also occur frequently in NEBC, with studies demonstrating prevalence of between 55 and 100% of SCNC cases [24]. Notably, small cell carcinomas originating from other sites, including lung, prostate, and skin, did not harbor *TERT* promoter mutations, highlighting its potential as a specific diagnostic biomarker for SCNC of the bladder.

Upfront RC was long considered the standard treatment for SCNC; however, outcomes were poor, as many patients were pathologically upstaged and subsequently experienced disease relapse and death within approximately 2 years of RC. New evidence from large-scale retrospective series and small clinical trials have demonstrated an overall and disease-free survival benefit with NAC in surgically resectable SCNC [22,27,28]. A large retrospective study of 172 MD Anderson patients with surgically resectable SCNC showed that those who received NAC (94% cisplatin-based) had a median OS of 159.5 months vs. 18.3 months with upfront cystectomy, and a 5-year DSS of 79% versus 20% (*p* < 0.001). NAC also resulted in substantial pathologic downstaging, with 62% of patients achieving ≤ pT1N0 disease at cystectomy compared with only 9% in the upfront surgery group (OR 44.55) [27]. Current AUA/ASCO/ASTRO/SUO consensus, as well as the National Comprehensive Cancer Network^®^ (NCCN^®^, Plymouth Meeting, PA, USA) guidelines, recommend concurrent chemoradiotherapy or platinum-based neoadjuvant chemotherapy followed by local consolidation (with chemoradiation or RC) for localized SCNC. Chemotherapy regimens have been extrapolated from evidence in the SCLC space and consist of etoposide–cisplatin or ifosfamide-based regimens [28,29,30].

Given that approximately 40% of SCNC cases exhibit mixed histologic features of small cell and conventional UC, the 2022 WHO classification (Table 1) recommends reporting the proportion of any neuroendocrine component because of its prognostic and therapeutic significance. Even focal neuroendocrine elements, driven by *TP53/RB1* co-inactivation and *ASCL1*-mediated transcriptional reprogramming, carry sufficient proliferative and metastatic capacity to dominate tumor behavior. This biological principle underpins the recommendation to treat any tumor harboring a small cell component with SCNC-directed therapy (etoposide–cisplatin or ifosfamide-based regimens) regardless of the proportion of neuroendocrine differentiation present. Standardized reporting thresholds defining the minimum clinically significant neuroendocrine proportion remain an important unresolved issue.

Unlike SCLC, which carries a high incidence of brain metastases (60%), SCNC has a relatively lower incidence (10%). Nonetheless, brain imaging is recommended in all patients with bladder cancer possessing any small cell component or neuroendocrine features [25,29]. Of note, some retrospective series with high-risk SCNC patients (pT3b+, N+, M+) have demonstrated lower rates of cranial metastases with prophylactic brain irradiation [31].

The role of immunotherapy in NEBC is under investigation. Genomic profiling of NEBC has shown that despite a high tumor mutational burden, its tumor microenvironment is predominantly immunologically “cold,” characterized by limited intratumoral T-cell infiltration [24,32]. The co-loss of TP53 and RB1 characteristic of SCNC may explain the immunologically cold tumor microenvironment given that RB1 loss has been linked to downregulation of antigen-presenting machinery (MHC-I expression) and TP53 loss impairs immune surveillance signaling [33]. This may provide a mechanistic basis for the limited clinical efficacy seen to date with immune checkpoint inhibitor (ICI) monotherapy and combined ICI in SCNC [32,34]. Combination strategies incorporating chemotherapy and immunotherapy appear to enhance immunogenicity by promoting immune infiltration and modulating the tumor microenvironment. A recent phase Ib clinical trial (NCT03582475) of pembrolizumab + platinum/etoposide in SCNC (n = 7) reported an overall response rate of 43% and a 12-month progression free survival rate of 86%, with one patient achieving pT0 at cystectomy [35]. However, it is important to consider that these conclusions should be limited regarding long-term efficacy given the small sample size (n = 7) and phase 1b safety endpoint nature of the study. Additional novel therapies should be evaluated through prospective clinical trials involving NEBC patients.

## 7. Sarcomatoid Subtype

Sarcomatoid urothelial carcinoma (SUC) is a rare and highly aggressive histologic subtype of bladder cancer accounting for approximately 0.1% to 0.3% of all bladder malignancies [36]. Two SEER database studies including patients with SUC (N = 569; N = 301) and conventional UC (N = 37,740; N = 46,515) demonstrated that SUC is associated with significantly shorter disease-specific survival, higher rates of extravesical invasion, and nodal metastasis. Median disease-specific survival for SUC is significantly shorter, at approximately 16 months compared 82 months for conventional UC. SUC has been associated with prior radiation or cyclophosphamide exposure [36,37,38].

SUC is characterized by mixed histopathologic morphology, demonstrating a combination of epithelial and mesenchymal differentiation driven by dysregulation of the EMT pathway. EMT occurs when epithelial cells lose their adhesive features and develop migratory infiltrative properties typically associated with mesenchymal spindle cells, a process commonly linked to increased invasiveness and metastatic potential [39]. SUC exhibits uniformly low mRNA expression levels of the luminal genes and is thought to develop from basal precursor urothelial carcinomas [34,35]. This is supported by several genomic studies including a large series of 136 patients with SUC, which showed that most tumors (75%) were positive for basal cell marker CK5/6 [38].

SUC has high overall tumor mutational burden. A comprehensive genomic analysis of 28 SUC cases by Guo et al. demonstrated that mutations in *TP53* (72%), *PIK3CA* (39%), and *RB1* (39%) occurred at significantly higher frequencies in SUC than in conventional UC (*p* < 0.01) [39,40]. In terms of targetable mutations, *PD-L1* expression and immune cell infiltration has been shown to be higher in the sarcomatoid component of SUC, therefore providing opportunities for immune checkpoint therapy in this subtype. A retrospective study of 755 patients treated with pembrolizumab found that patients with sarcomatoid subtype achieved significantly better outcomes than those with conventional UC: objective response rate of 36.8% vs. 24.5% (*p* = 0.031), disease control rate of 52.6% vs. 36.7% (*p* = 0.032), and superior overall survival (HR 0.37, 95% CI 0.15ߝ0.90; *p* = 0.023) [41]. Additionally, a single-arm phase 2 analysis demonstrated 75% (6/8 patients) pathologic complete response in patients receiving neoadjuvant atezolizumab prior to RC in SUC [42].

Despite these findings, large-scale evidence for the use of perioperative immunotherapy in SUC is currently lacking. Contemporary landmark trials (NIAGARA trial, KEYNOTE-905) did not report sarcomatoid-specific subgroup analyses; therefore, most experts continue to favor offering NAC to cisplatin-eligible patients with SUC. Two large analyses from the NCDB found that NAC was associated with pathological downstaging and improved OS for patients with SUC [42,43]. However, the survival benefit of NAC in SUC is controversial given that other studies have failed to demonstrate a statistically significant OS benefit [44].

## 8. Squamous Differentiation and Pure Squamous Cell Carcinoma

### 8.1. UC with Squamous Differentiation

UC with squamous differentiation (SqD) is by far the most common SBC, occurring in up to 60% of urothelial carcinoma cases [45]. The 2022 WHO classification categorizes SqD under conventional UC that has undergone divergent differentiation acquiring morphologic features of squamous carcinoma, histologically characterized by the presence of intercellular bridges and keratinization [46,47]. The percentage of squamous component should be reported.

Immunohistochemically, these tumors frequently co-express urothelial markers such as S100P, GATA3 and uroplakin III, alongside squamous-associated markers including cytokeratin 14 (CK14) and desmoglein-3 [48]. This overlapping protein expression profile reflects the hybrid biology of these tumors, in which areas of morphologic squamous differentiation may still demonstrate urothelial marker expression and vice versa.

The treatment of SqD resembles that of conventional UC, including eligibility for neoadjuvant cisplatin-based chemotherapy and immunotherapy. In contrast to conventional UC, SqD is linked to advanced pathological stage and worse oncologic outcomes [47]. In a retrospective cohort of 71 patients with SqD, the pathologic complete response rate at cystectomy reached showed 60% among those who received NAC, compared with 13.7% in patients who did not (*p* < 0.001). This advantage was most pronounced when squamous differentiation accounted for less than 50% of the tumor [49].

### 8.2. Pure Squamous Cell Carcinoma

Pure squamous cell carcinoma of the bladder (SCC) is histologically distinct from urothelial carcinoma. Pure SCC is diagnosed when there is no identifiable UC component, and it constitutes 1.2–4.5% of all bladder cancers in Western regions. In regions where bilharzial Schistosoma infection is endemic (Africa, Middle East, Southeast Asia, South America), pure SCC accounts for up to 75% of bladder cancer cases. Lymph node metastasis is uncommon with bilharziasis because of extensive associated mural fibrosis; therefore, it has been associated with relatively good prognosis [50]. Long-term indwelling catheterization, recurrent urinary tract infections, and neurogenic lower urinary tract disease are the conditions most frequently liked to non-bilharzial SCC. Non-bilharzial SCC is recognized as a highly aggressive tumor entity and an independent predictor of cancer-specific mortality with advanced stages [50,51]. In a comparison of patients with bilharzial SCC and SqD, those with SqD demonstrated reduced 5-year RFS, and a greater likelihood of extravesical extension (58.3% vs. 46.2%: *p* = 0.006) and nodal metastatic disease (34.5% vs. 14.5%: *p* < 0.0001) relative to the bilharzial SCC group [47].

With regard to IHC, pure SCC of the bladder demonstrates a complete shift toward squamous lineage commitment, characterized by diffuse expression of squamous markers such as CK14 and desmoglein-3 and absence of urothelial markers including GATA3 and uroplakin III, with only rare or focal S100P expression [48]. This distinct immunophenotype supports the concept that pure squamous cell carcinoma represents a biologically separate entity from SqD, which may underlie its reduced responsiveness to systemic therapies typically used for UC.

Psoriasin, a calcium-binding protein expressed by squamous epithelial cells, demonstrates increased expression in SCC. Its presence in urine suggests that it could serve as a valuable biomarker to help clinicians identify this subtype [50,51]. Gene expression profiling studies have demonstrated that pure SCC shows abundant *EGFR* expression and represents a potential future therapeutic target for patients with SCC [52].

Currently there is no proven role for neoadjuvant nor adjuvant chemotherapy for pure SCC, and treatment relies on surgery or chemoradiotherapy, with clinical trials preferred for advanced disease [29]. RC has been established as an effective treatment, though many patients present with advanced, unresectable disease. In a SEER-based analysis of 5653 patients with non-bilharzial SCC, outcomes were poor (median survival 13 months; 5-year OS 28%), underscoring the aggressive nature of this disease. Surgical management was the only modality associated with improved survival, with a 5-year OS of 40% compared to 21% without surgery (*p* < 0.01), and superior outcomes with surgery alone (35%) versus multimodal approaches [53].

## 9. Conclusions

SBC represent a biologically and clinically heterogeneous group of tumors characterized by distinct molecular features that underlie their aggressive phenotypes and variable therapeutic responses. This review provides a comprehensive summary of the major subtypes of bladder cancer, including their epidemiology, molecular alterations, clinical behavior, and therapeutic implications (Table 2).

Despite growing recognition of their clinical importance, several fundamental challenges continue to limit optimal management of SBC. Diagnostic reproducibility remains unresolved, with interobserver variability documented in several subtypes and ongoing challenges in distinguishing histologic subtypes from divergent differentiation. Standardized diagnostic criteria, mandatory subtype proportion reporting, and review by genitourinary subspecialty-trained pathologists are essential steps toward consistent classification.

The benefit of neoadjuvant chemotherapy remains uncertain across several subtypes. These uncertainties are compounded by the persistent underrepresentation of histologic subtypes in prospective trials which have not reported subtype-specific analyses. Prospective trials must incorporate subtype stratification to generate necessary, high-level, subtype-specific evidence.

Finally, while molecular profiling has identified promising therapeutic targets across subtypes, their utility as predictive biomarkers remains largely unvalidated. Biomarker-driven trial designs incorporating correlative molecular analyses will be essential to translate these discoveries into actionable, precise treatment strategies for this high-risk patient population.

## 10. Future Directions

To tailor treatment algorithms to various subtypes of bladder cancer, molecular profiling across multicohort studies should be prioritized. Ultimately, this profiling can help examine the prognostic differences across SBC and even within individual subtypes to help identify genomic targets and markers to help select those that may need more aggressive treatment. Moreover, it is important to note that much of the evidence discussed comes from retrospective series, NCDH/SEER analysis, and overall small cohort studies, much of which are inherently vulnerable to selection bias, missing data, and incomplete molecular annotation. Therefore, further treatment and molecular characterizations should be augmented with larger scale analyses and clinical trials. Additionally, future clinical trials involving novel treatments such as immunotherapies in muscle invasive disease should include SBC to help determine the impact of SBC identification on efficacy of these novel treatments.

## Figures and Tables

**Table 1:** WHO classification of tumors of the urinary tract, 5th edition, 2022. The 2022 WHO classification of urinary tract tumors continues to rely primarily on histomorphology for classifying urothelial tumors while also incorporating emerging molecular insights. The classification addresses challenges with intratumoral heterogeneity, recommending papillary tumors be classified as high-grade if ≥5% of the lesion contains high grade features. The importance of reporting the proportion of neuroendocrine component is highlighted given its prognostic and therapeutic significance, particularly regarding recommendation for chemotherapy. Several previously recognized entities, including “urothelial hyperplasia” and “urothelial proliferation of uncertain malignant potential,” have been removed or redefined. Of note, SBC are all considered high-grade tumors, even subtypes with a bland morphology, such as nested-type or microcystic-type urothelial carcinomas [4].

**Urothelial tumors** *Non-invasive urothelial neoplasms* *Invasive urothelial neoplasms* Invasive urothelial carcinoma Infiltrating urothelial carcinoma with squamous differentiation Infiltrating urothelial carcinoma with glandular differentiation Micropapillary urothelial carcinoma Plasmacytoid urothelial carcinoma Sarcomatoid urothelial carcinoma Nested urothelial carcinoma Tubular and microcystic urothelial carcinoma Lymphoepithelioma-like urothelial carcinoma
**Squamous cell neoplasms of the urinary tract** *Squamous cell papilloma of the urinary tract* *Squamous cell carcinomas of the urinary tract* Verrucous carcinoma of the bladder Pure urothelial squamous cell carcinoma
**Neuroendocrine Tumors** Small cell neuroendocrine carcinoma Large cell neuroendocrine carcinoma Well differentiated neuroendocrine tumor Paraganglioma
**Glandular neoplasms** *Adenomas* Villous adenoma *Adenocarcinomas* Adenocarcinoma NOS Urachal and diverticular neoplasms Urachal carcinoma Diverticular carcinoma
**Urethral neoplasms** *Urethral accessory gland carcinomas*
**Mesenchymal Tumors**
**Tumors of Müllerian type** Clear cell adenocarcinoma of the urinary tract Endometrioid carcinoma of the urinary tract

**Table 2:** OS and survival estimates are derived from retrospective series and national database analyses and should be interpreted with caution given inherent selection bias and variability in study populations. Trial references are provided in short format; refer to reference list for full citations. ICI: immune checkpoint inhibitor; IO: immunotherapy; DDR: DNA damage response; pCR: pathologic complete response; CSS: cancer-specific survival.

Subtype	Overall Survival (OS)	Key Molecular/IHC Features	Treatment Response and Clinical Implications
MPC	Median OS ~40 months following RC	TP53, TERT, ARID1A, ERBB2, RB1 mutations; ERBB2 amplification/HER2 overexpression linked to worse OS	NMIBC: Early RC favored over BCG; 5-year CSS 100% with RC vs. 60% with BCG [14]. MIBC: NAC associated with improved pT0 rates (52% vs. 19%) [15] and pathologic downstaging [6] though consistent OS benefit unproven; NAC pCR 45% vs. 13% upfront RC [16]; HER2-directed therapy under investigation.
PCUC	Median OS ~15–18 months, ~30% at follow up, poor prognosis	CDH1 loss/hypermethylation → E-cadherin loss; TP53, FGFR3, RB1, PIK3CA, TERT; mTOR pathway activation; HER2 (25–83%), Nectin-4 positivity (~63%)	MIBC: NAC not associated with consistent survival benefit [7,8]; early RC recommended; salvage immunotherapy associated with reduced cancer-specific death risk (HR 0.11) [8]. Nectin-4 (enfortumab) and HER2 (trastuzumab) potential targets
AC	5-yr OS 36%	Primary AC: TP53, KRAS, PIK3CA (colorectal-like); urachal AC: SMAD4 (24%); pure AC rarely has DDR mutations; UC w/glandular diff. resembles pure UC genomically	UC with glandular diff.: Treated as conventional UC; NAC associated with pathologic downstaging (pT0N0 17% vs. 25% pure UC) [20]; NAC reduced non-organ-confined disease without OS prolongation [22]; Pure AC/Urachal AC: No established NAC role; surgery is mainstay; clinical trials preferred for advanced disease
SCNC	Median OS ~11–16 months (all stages), 18–40 months	TP53 and RB1 co-inactivation (~80%); MHC-I downregulation → immunologically “cold”; TERT promoter (55–100%) bladder-specific	NAC (cisplatin/etoposide) followed by RC or chemoradiation strongly recommended, median OS 159.5 vs. 18.3 mo w/upfront RC [27]; Brain imaging recommended; ICI monotherapy limited, chemo + IO promising [35]
SUC	Median OS ~16–30 months	EMT dysregulation; basal phenotype (CK5/6+ in ~75%); TP53 (72%), PIK3CA (39%), RB1 (39%); high TMB; elevated PD-L1	NAC associated with downstaging [43,44] but survival benefit controversial [45]; Pembrolizumab ORR 36.8% vs. 24.5% in UC, superior OS (HR 0.37) [41].
SqD	Present at higher pathological stage, when stage-matched OS is comparable to conventional AC	Co-expression of urothelial (GATA3, uroplakin III, S100P) and squamous markers (CK14, desmoglein-3)	Treated like UC; NAC pCR 60% vs. 13.7% without NAC, benefit greatest when squamous component <50% [49]
SCC	Median OS ~13 months (non-bilharzial); 5-year OS 28%; bilharzial SCC	Full squamous commitment: CK14, desmoglein-3 positive; GATA3/uroplakin III negative; EGFR overexpression; Psoriasin (urinary biomarker)	No proven role for NAC; RC is mainstay (5-yr OS 40% w/surgery vs. 21% w/o) [53]; EGFR-directed therapy under investigation

## Data Availability

No new data were created or analyzed in this study. Data sharing is not applicable to this article.

## Abbreviations

The following abbreviations are used in this manuscript:

BC	Bladder cancer
BCG	Bacillus Calmette–Guérin
CK14	Cytokeratin 14
CK5/6	Cytokeratin 5/6
CI	Confidence interval
CSS	Cancer-specific survival
DDR	DNA damage response
EGFR	Epidermal growth factor receptor
EMT	Epithelial-to-mesenchymal transition
GATA3	GATA binding protein 3
HGT1	High-grade T1
HR	Hazard ratio
ICI	Immune checkpoint inhibitor
IHC	Immunohistochemistry
IO	Immunotherapy
MFS	Metastasis-free survival
MIBC	Muscle invasive bladder cancer
MPC	Micropapillary subtype carcinoma
MPUC	Micropapillary urothelial carcinoma
MSKCC	Memorial Sloan Kettering Cancer Center
MVUC	Muscle-invasive variant urothelial carcinoma
mRNA	Messenger RNA
NAC	Neoadjuvant cisplatin-based chemotherapy
NCDB	National Cancer Database
NMIBC	Non-muscle invasive bladder cancer
OS	Overall survival
PD-L1	Programmed death-ligand 1
PIK3CA	Phosphatidylinositol-4,5-bisphosphate 3-kinase catalytic subunit alpha
PCUC	Plasmacytoid variant
RB1	Retinoblastoma 1
RC	Radical cystectomy
RFS	Recurrence-free survival
S100P	S100 calcium-binding protein P
SBC	Histoligical subtype bladder cancer
SCC	Squamous cell carcinoma
SEER	Surveillance, Epidemiology, and End Results
SqD	Squamous differentiation
SUC	Sarcomatoid urothelial carcinoma
SUO	Society of Urologic Oncology
TERT	Telomerase reverse transcriptase
TP53	Tumor protein p53
UC	Urothelial carcinoma
UTI	Urinary tract infection
WHO	World Health Organization
